# Helical tomotherapy with concurrent capecitabine for the treatment of inoperable pancreatic cancer

**DOI:** 10.1186/1748-717X-5-60

**Published:** 2010-06-28

**Authors:** Jeong-Seon Ji, Chi-Wha Han, Jeong-Won Jang, Bo-In Lee, Byung-Wook Kim, Hwang Choi, Ji-Yoon Kim, Young-Nam Kang, Chul-Seung Kay, Ihl-Bohng Choi

**Affiliations:** 1 Department of Internal Medicine, The Catholic University of Korea, Incheon St. Mary's Hospital, 665, Bupyung 6-dong, Bupyung-gu, Incheon, 403-720, Republic of Korea; 2 Department of Internal Medicine, The Catholic University of Korea, St Mary's Hospital, 62, Youidodong, Youngdeoungpogu, Seoul, 150-713, Republic of Korea; 3 Department of Radiation Oncology, The Catholic University of Korea, St Mary's Hospital, 62, Youidodong, Youngdeoungpogu, Seoul, 150-713, Republic of Korea; 4 Department of Radiation Ocology, The Catholic University of Korea, Seoul St. Mary's Hospital, 505 Banpo-dong, Seocho-gu, Seoul 137-040, Republic of Korea; 5 Department of Radiation Oncology, The Catholic University of Korea, Incheon St. Mary's Hospital, 665, Bupyung 6-dong, Bupyung-gu, Incheon, 403-720, Republic of Korea; 6 Cyberknife Clinic, Wooridul Spine Hospital, 47-4, Chungdamdong, Kangnamgu, Seoul, Republic of Korea

## Abstract

**Background:**

Helical tomotherapy, an advanced intensity-modulated radiation therapy with integrated CT imaging, permits highly conformal irradiation with sparing of normal tissue. Capecitabine, a pro-drug of 5-FU that induces thymidine phosphorylase can achieve higher levels of intracellular 5-FU when administered concurrently with radiation. We evaluated the feasibility as well as the clinical outcome of concurrent administration of capecitabine with tomotherapy in patients with advanced pancreatic cancer.

**Methods:**

Nineteen patients with advanced pancreatic cancer including primarily unresectable disease and recurrence after curative surgery were included in the study. Two planning target volumes (PTV) were entered: PTV1 is gross tumor volume; and PTV2, the volume of the draining lymph nodes. The total doses to target 1 and target 2 were 55 and 50 Gy, respectively. Capecitabine at 1600 mg/m^2^/day was administered on each day of irradiation.

**Results:**

Twenty six measurable lesions were evaluated. Overall in-field response rate was 42.3%; partial responses were achieved in 53.3% of the pancreatic masses, 28.6% of distant metastatic lesions and 25.0% of regional lymph nodes. The median duration of follow-up after tomotherapy was 6.5 months. None of the lesions showed in-field progression. Treatment was well tolerated with only minor toxicities such as grade 1 nausea (one patient), grade 1 hand-foot syndrome (one patient) and grade 1/2 fatigue (three patients).

**Conclusions:**

Helical tomotherapy with concurrent capecitabine is a feasible option without significant toxicities in patients with advanced pancreatic cancer. We achieved excellent conformal distribution of radiation doses and minimal treatment-related toxicities with promising target volume responses.

## Background

Surgical resection is the standard treatment for localized non-metastatic pancreatic cancer. Data from the Surveillance Epidemiology and End Results (SEER) registry indicate that only about 10% of cases are able to undergo surgery with curative intent, and only a very small number of those are cured because of the high incidence of local relapse and early metastases [1]. Many clinical trials have been carried out using chemotherapy with or without radiation therapy following curative surgical resection, with the aim of preventing local and distant recurrence. With the exception of gemcitabine, neither chemotherapy nor radiation improved survival [2]. For those with locally advanced unresectable or metastatic disease, systemic chemotherapy remains the principal means of improving survival or alleviating cancer-related symptoms

The radiation-sensitive structures in the upper abdomen (small intestine, stomach, kidneys, liver, and spinal cord), prevent conventional radiation therapy to the pancreas or to the pancreatic bed from delivering adequate doses, and irradiation is usually accompanied by severe gastrointestinal intolerance [3]. This may explain in part the absence of survival benefit in patients with locally advanced pancreatic cancer who receive radiation therapy alone. However, 5-FU-based concurrent chemoradiation yields modest survival benefits in patients with locally advanced unresectable pancreatic cancer [4,5]. Despite these findings, survival from pancreatic cancer is still poor, with approximately 23% of patients alive 12 months following diagnosis, and 5% alive at 5 years [1]. New radiation techniques including intensity modulated radiation therapy (IMRT), image guided radiation therapy (IGRT) and stereotactic radiosurgery make it possible to deliver optimally high doses to the target volume with minimal effect on adjacent radiosensitive tissues [6,7]. Helical tomotherapy is a sophisticated image-guided IMRT based on the ring gantry concept, employing a combination of a megavoltage CT scanner and a linear accelerator [8,9]. Capecitabine, a prodrug of 5-FU, is absorbed inert from the gastrointestinal tract and selectively metabolized to 5-FU in tumor cells. This selective conversion achieves higher levels of 5-FU in the tumor cells than can be obtained by intravenous administration of 5-FU. Additionally, radiation can magnify the tumor selectivity of capecitabine by upregulating thymidine phosphorylase in the tumor cells [10]. Capecitabine also acts as a radiation sensitizer by disturbing tumor cell DNA synthesis [11].

In this paper, we report our experience of concurrent administration of capecitabine with helical tomotherapy in patients with inoperable or recurrent pancreatic cancer. We achieved a highly conformal distribution of radiation doses and minimal treatment-related toxicities with excellent target volume responses.

## Methods

### Patient population

Between October 2005 and February 2008, nineteen patients with pancreatic cancer were treated with concurrent chemoradiation using helical tomotherapy and capecitabine. They included patients with locally advanced and unresectable disease, and those with local relapse following curative resection or with metastatic disease. Patients who were older than 18 years, who understood the written informed consent document and who were willing to sign it, were eligible for inclusion. The medical records of these patients were reviewed retrospectively. This review was approved by the hospital institutional ethical committee, and written informed consent was obtained from each patient.

### Radiotherapy

**Figure 1:**
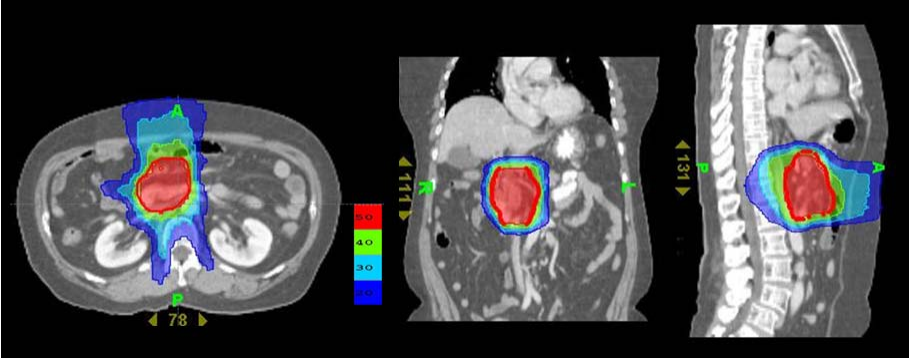
**Distribution of isodoses in the planning of helical tomotherapy in patients with advanced pancreatic cancer; axial (left), coronal (center) and saggital (right) representations**. Dose displayed in Gy. The different doses are represented by different colors. Red represents the target volume dose.

**Figure 2:**
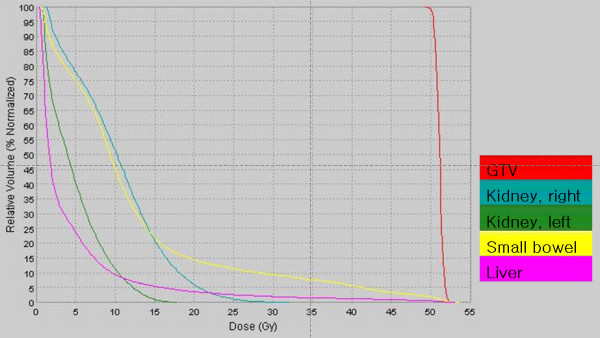
**Average dose-volume histogram for GTV and organs at risk**. Patients were prescribed doses of 55 Gy to PTV1 and 50 Gy to PTV2. GTV = gross tumor volume, PTV = planning target volume.

Radiotherapy was provided by helical tomotherapy (Tomotherapy Incorporated, Madison, WI, USA). Two planning target volumes (PTV) were entered for each patient [3]. PTV1 consisted of the gross tumor volume (GTV) as determined by CT scan, or the tumor bed (in post-surgical cases). PTV2 consisted of the draining lymph nodes, comprising the nodes in the porta hepatis, celiac axis, superior mesenteric and retroperitoneal areas. PTV2 extended 2 cm below the target volume and did not have to include the inferior mesenteric nodes. Both targets were treated simultaneously in 25 daily fractions, 5 days a week. Helical tomotherapy delivered 55 Gy to PTV1 and 50 Gy to PTV2. In some patients with distant metastases (liver or lung), the metastatic lesions were also targeted as another PTV. The distribution of isodoses in the helical tomotherpy treatment planning is shown in Figure 1. The dose and volume constraints for the normal structures are listed in Table 1. Figure 2 is an average delivered dose-volume histogram for GTV and organ at risk. Capecitabine (Xeloda; Roche Pharmaceuticals, Nutley, NJ) was given at 1600 mg/m^2^/day in two doses on each day of radiation and continued for the duration of the radiation therapy [3].

**Table 1:** Dose and volume constraints for organs at risk.

Structure	Maximum dose constraint (Gy)	Volume above limit (%)	Maximum dose (Gy)	Minimum dose (Gy)
Liver	45.00	10.00	52.83	0.30
Right kidney	1.00	1.00	20.60	0.38
Left kidney	15.00	20.00	20.57	0.54
Small bowel	45.00	10.00	53.34	0.18
Stomach	50.00	10.00	52.95	0.44
Duodenum	10.00	1.00	14.07	0.60

### Toxicity assessment

Acute toxicity (occurring within 90 days of radiotherapy) was scored using the National Cancer Institute Common Toxicity Criteria (NCI CTC), version 2, morbidity scales [12]. Late toxicity was scored using the Radiation Therapy Oncology Group (RTOG) scale for late toxicity [13]. Patients were evaluated on a weekly basis.

### Response assessment

The response of each targeted lesion (defined as the in-field tumor response) was evaluated by comparing, by the RECIST criteria, tumor size in pre- and post-treatment CT images 8 weeks after completion of concurrent chemoradiation therapy (CCRT). Two different radiologist evaluated the response rate.

### Statistical methods

All statistics are descriptive. Survival was compared using the Kaplan-Meier method. Statistical analyses were performed using SPSS software, version 15.0, Chicago.

## Results

### Patient and tumor characteristics

The patient characteristics are shown in Table 2. Twelve were male and seven were female. Median age was 64.0 (range, 46 - 83). Median duration from diagnosis to CCRT was 1.5 months (range, 0.2 - 63.3). The patients were classified with respect to disease status as follows: 1) eight had primarily unresectable disease without metastasis, and no history of previous treatment, 2) three had local relapse following complete resection, and 3) eight had metastatic disease in the liver, lung or peritoneum (three had metastases on first diagnosis and five had metastases that developed during the course of disease). Eight patients had previously received systemic chemotherapy.

**Table 2:** Patient and tumor characteristics

Patient	Sex	Age	Primary tumor site	Previous operation	Previous chemotherapy	TNM (stage)	Duration of follow-up after diagnosis (months)	Site of metastasis	Site of tomotherapy
1	F	53	Head	No	No	T4N1M0(IVA)	2.9		Pancreas
2	M	61	Body	Yes	Gemcitabine #6, Cisplatin/Capecitabine	T3N0M0 (II)	4.9		Pancreas
3	M	67	Tail	No	No	T4N1M0(IVA)	1.5		Pancreas
4	F	76	Body	No	Gemcitabine #5	T4N1M0(IVA)	7.6		Pancreas
5	M	57	Body	No	Gemcitabine/Capecitabine	T4N1M1(IVB)	1.2	Liver	Pancreas
6	F	64	Body, tail	No	No	T4N1M0(IVA)	0.2		Pancreas
7	M	67	Body	No	No	T4N1M0(IVA)	1		Pancreas
8	F	71	Body	No	Gemcitabine/Cisplatin #3	T3N1M1(IVB)	8	Liver	Pancreas
9	M	46	Body, tail	No	No	T4N1M0(IVA)	0.7	Peritoneum	Pancreas
10	F	80	Body, tail	No	No	T3N1M0(III)	2.3		Pancreas
11	F	64	Tail	No	Gemcitabine/Cisplatin #1	T4N1M1(IVB)	1.3	Liver	Pancreas, Liver
12	M	59	Head	No	No	T3N1M1(IVB)	1.4	Liver	Pancreas
13	M	68	Body, tail	No	No	T4N1M1(IVB)	0.6	Liver	Pancreas, Liver
14	F	54	Neck, body	No	Gemcitabine/5 - FU #2	T3N1M0(III)	5.5		Pancreas
15	M	57	Body	Yes	Gemcitabine, Cisplatin/5 FU #6	T4N1M1(IVB)	5.2	Liver	Pancreas
16	M	83	Head	No	No	T4N1M0(IVA)	0.2		Pancreas
17	M	54	Head	No	No	T4N1M0(IVA)	0.2		Pancreas
18	M	64		Yes	Gemcitabine/xeloda #9, Irinotecan #2	M1(IVB)	63.3	Lung	Lung
19	M	58	Head	No	No	T4N1M0(IVA)	2.4		Pancreas

### In-field tumor responses

**Figure 3:**
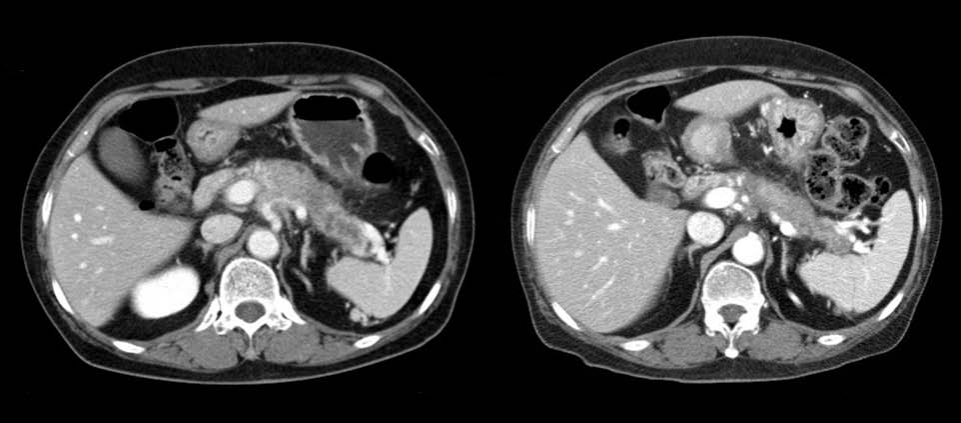
**Abdomenal CTs before (left) and after (right) helical tomotherapy with concurrent capecitabine**. Two months after helical tomotherapy the volume of the pancreatic tumor is significantly reduced.

Twenty six lesions were targeted in nineteen patients (Table 3). They included 15 pancreatic masses, 4 regional metastatic lymph nodes and 7 distant metastatic lesions. Of the 15 pancreatic masses, 8 showed partial responses (PR, 53.3%) and 7 stable disease (SD, 46.6%). Of the 4 regional metastatic lymph nodes, one showed PR (25.0%) and three, SD (75.0%). Of the seven distant metastatic lesions (six hepatic metastases and one pulmonary metastasis), 2 (a pulmonary lesion and a hepatic lesion) showed PR (28.6%) and 5, SD (71.4%). Although there were no complete responses (CR), the overall response rate was 42.3%. It is of interest that no target lesions showed in-field progression during the observation period. Figure 3 illustrates a typical case of a pancreatic lesion treated with CCRT.

**Table 3:** In-field tumor response rates of the target lesions after tomotherapy and concurrent capecitabine treatment

Target lesions	CR	PR	SD	PD
Pancreatic mass (n = 15)	0 (0)	8 (53.3)	7 (46.7)	0 (0)
Regional lymph nodes (n = 4)	0 (0)	1 (25.0)	3 (75)	0 (0)
Distant metastasis (n = 7)	0 (0)	2 (28.6)	5 (71.4)	0 (0)
Liver (n = 6)	0 (0)	1 (16.7)	5 (83.3)	0 (0)
Lung (n = 1)	0 (0)	1 (100)	0 (0)	0 (0)
Overall (n = 26)	0 (0)	11 (42.3)	15 (57.7)	0 (0)

CR, complete response; PR, partial response; SD, stable disease; PD, progressive disease

Numbers in parentheses are percentages

### Prognosis and survival

The median duration of follow-up after CCRT was 6.5 months (range, 1.1-17.6, Table 4). The one-year survival rate was 36.8%, and median survival time was 6.5 months (range 1.1-21.0). The median survival time in group I (patients with locally advanced disease without metastases) was 9.25 months (range, 2-18.4, Table 5). In comparison with patients who had locally advanced and unresectable disease without metastases or a previous chemotherapy history, the others (those who had metastases at the time of CCRT, and a case with local relapse after previous curative surgery as well as those with a history of previous chemotherapy) showed poor survival (*p *= 0.063); 4.4 months (range, 1.1-21) versus 12.55 months (range, 6.5-18.4). Of the patients in group I, those who had no history of previous chemotherapy survived better than those with a history of previous chemotherapy (*p *= 0.0009); 12.55 months (range, 6.5-18.4) versus 3.9 months (range, 2-5.8).

**Table 4:** Clinical outcomes in the nineteen patients treated with tomotherapy and concurrent capecitabine

Patient	Overall In-field tumor response	Duration of tumor response (months)	Treatment-related toxicity	Duration of follow-up after tomotherapy (months)	Out-field progression state	Cause of death other than cancer progression	Duration of survival after tomotherapy (months)
1	Stable disease	5.9	fatigue (grade 2)	7.3	Progressed		7.3
2	Partial response		hand foot syndrome (grade 1)	3.2			4.8
3	Stable disease			10.7			11.2
4	Stable disease	3.8		5.8	Progressed		5.8
5				1.1		Pneumonia	1.1
6	Partial response		fatigue (grade 2)	6.8			13.9
7	Stable disease	6.9		15	Progressed		16.3
8				1.8			1.9
9	Partial response	2.4	nausea (grade 1)	4.4	Progressed		4.4
10	Partial response			13.6			13.9
11	Stable disease	4.1	fatigue (grade 1)	4.1	Stable disease	Pulmonary thromboembolism	4.1
12	Stable disease	2.2		3.4	Progressed		3.9
13	Stable disease	2.5		6.5	Progressed		6.5
14				2		DUB, Pneumonia	2
15	Stable disease			10.5			10.5
16	Partial response	7.3		7.3	Stable disease	Pneumonia	7.3
17	Partial response			14.9			18.4
18	Partial response			17.6			21
19	Partial response	3.2		6.2	Progressed		6.5

DUB, duodenal malignant ulcer bleeding

**Table 5:** Survival of pancreatic cancer patients treated with tomotherapy and concurrent capecitabine

Group	Characteristics	Median duration of survival (months)
I	Locally advanced without metastasis (n = 10)	9.25 (2.00-18.4)
	No previous chemotherapy (n = 8)	12.55 (6.50-18.4)
	Previous chemotherapy (n = 2)	3.90 (2.00, 5.8)

II	Locally relapsed without metastasis following complete resection (n = 1)	4.80 (4.80)

III	Metastatic disease (n = 8)	4.25 (1.10-21.00)
	De novo (n = 3)	4.40 (3.90-6.50)
	Relapsed (n = 5)	4.10 (1.10-21.00)

Data in parentheses are ranges of survival times

Progression of disease outside the targeted tumor volume (defined as the out-field progression) occurred in 7 patient. The median time to out-field progression was 3.8 months (range 2.2-7.3) with or without systemic chemotherapy following CCRT.

### Toxicity

Acute toxicity is summarized in Table 6. As shown, only minor toxicities developed. The most common acute toxicity was grade 1 or 2 fatigue that occurred 2 to 3 weeks after the start of tomotherapy (three patients, 16.7%). Intriguingly, no treatment was interrupted due to gastrointestinal side effects. Only grade 1 nausea developed in one patient (5.6%). Grade 1 hand-foot syndrome related to oral capecitabine also developed in one patient (5.6%). None experienced hematologic toxicities during the treatment. All toxicities were manageable medically and regressed spontaneously, and they did not interfere with the scheduled radiotherapy. There were no treatment-related deaths and no grade 3 or 4 toxicity. Therefore, treatment was well tolerated by all patients.

**Table 6:** Treatment-related toxicity

	Grade 1	Grade 2
Fatigue	1 (5.6)	2 (11.1)
Nausea	1 (5.6)	0 (0)
Hand-foot syndrome	1 (5.6)	0 (0)

Data in parentheses are percentages

## Discussion

The majority of pancreatic cancer patients have advanced disease at the time of diagnosis due to a lack of symptoms and signs. Without treatment, mean survival time is 4-6 months and overall 5-year survival remains less than 5%. [14]. The only curative option is surgery, but only 10-20% of patients have tumors appropriate for radical resection [15]. Advanced pancreatic cancer is generally incurable and all therapies have significant limitations. The response to systemic chemotherapy is poor, with an approximately 20% response rate. The conventional radiation dose to the tumor volume is not large enough to cure patients because pancreatic tumors move markedly as patients breathe, and are surrounded by the duodenum, which is the dose-limiting organ [16]. Compared with chemotherapy alone or radiotherapy alone, chemoradiotherapy prolongs median survival somewhat, to approximately 9-12 months, in those with locally advanced unresectable disease [5].

Helical tomotherapy, a new radiotherapy system, is a helical IMRT with integrated CT imaging, offering highly conformal radiation with normal tissue sparing. The basis of image guidance is utilizing daily images gained in the treatment position in order to visualize daily organ variations and setup errors [17-19]. The radiation is discharged as a fan beam by a linear accelerator mounted on a turning gantry and is adjusted by a rapid pneumatically driven binary slit collimator [20]. The speed of gantry rotation and table movement is uniform for the entire fraction. Hence helical tomotherapy can provide significant conformal dose distributions at numerous locations [21-24].

Helical tomotherapy can treat multiple lesions more rapidly than conventional radiotherapy, for which multiple target points are necessary [20]. Moreover it is an ideal device for delivering multifocal, high-dose radiation without a significant increase in toxicity [9,25]. Thus it allows us to treat patients with multiple targets including metastatic lesions.

The ideal concurrent chemotherapeutic agent in the therapy of pancreatic cancer should have both a systemic effect and radiosensitizing properties [16]. Capecitabine has a pronounced radiosensitizing effect on tumor cells such that DNA strand breakage induced by radiation is more difficult to repair [11]. The regimen described here takes advantage of the tumor-selective ability of capecitabine to enhance radiation effects within the tumor but not in the surrounding normal tissues. This can be ascribed to a higher 5-FU concentration in tumor cells and the induction of thymidine phosphorylase by the irradiation [10]. Also, the use of capecitabine is attractive because it is absorbed as an inert drug, causing little direct toxicity in the gastrointestinal tract.

Ben-Josef et al [3] treated 15 patients with unresectable or recurrent pancreatic cancer with IMRT and concurrent capecitabine. In that study, the regimen was well tolerated without significant toxicities, and efficacy was encouraging.

Another basis for offering radiotherapy to patients with pancreatic cancer is palliation of symptoms due to local invasion, such as biliary and gastrointestinal obstruction [26]. The drawbacks of radiotherapy include the acute and chronic toxicities of radiotherapy, particularly when the indication is palliation. Because of its ability to restrict the dose to normal organs and minimize radiation toxicities, helical tomotherapy may be an ideal palliative option for challenging cases of pancreatic cancer [27].

In our study, the overall in-field tumor response rate was 42.3%. Previous studies have reported 10-50% response rates for locally advanced pancreatic cancer with chemoradiotherapy [28-32]. The high response rate in our study is due to in-field assessment of responses. Considering the advanced stage of our patients, the in-field response rate is encouraging. It may be possible to increase this response rate by increasing the dose of capecitabine.

It may be noted that helical tomotherapy with concurrent capecitabine yielded excellent disease control within the radiation field, with an in-field disease control rate of essentially 100%. This could be thought to be a significant therapeutic benefit.

Median overall survival after tomotherapy was only 6.5 months. This was because of advanced stages of our study population (tumor stages III or IV). Our study included patients with locally advanced disease, local relapse following complete resection, and metastases.

Patients who had locally advanced disease without metastasis or a previous history of chemotherapy showed a tendency to survive longer than the others (12.55 versus 4.4 months) after tomotherpy. In our opinion, tomotherapy with concurrent capecitabine should be the first option for inoperable pancreatic cancer, especially in patients without metastases or a previous history of chemotherapy.

Although our patients were elderly, with a median age of 64, treatment was well tolerated. The majority of treatment-related toxicities were mild and transient. Only grade 1/2 fatigue, nausea and hand-foot syndrome developed, and they subsided with symptomatic care and without prematurely stopping radiotherapy. There was no direct treatment-related grade 3/4 toxicity or death. Therefore helical tomotherapy is a safe option in the treatment of advanced pancreatic cancer.

This study had several limitations. First, the number of cases was low. Second, the heterogeneity of the study population made direct comparison with other studies difficult. Third, long term treatment effects and late toxicities remain to be evaluated because the median follow-up time in our study was relatively short.

Although there was no in-field progression during the observation period, out-field progression occurred in seven patients. This observation provides a rationale for follow-up systemic chemotherapy after tomotherapy to prevent or delay out-field progression. Hence, subsequent chemotherapy such as gemcitabine alone or erlotinib combined with gemcitabine should be performed in eligible patients [33,34].

There are only two examples of the clinical application of helical tomotherapy for locally advanced pancreatic cancer [35]. To the best of our knowledge, this is first comprehensive analysis of the clinical application of helical tomotherapy for a group of inoperable or recurrent pancreatic cancers.

## Conclusions

Our study demonstrates that helical tomotherapy with concurrent capecitabine is a feasible and safe option for locally advanced unresectable or metastatic pancreatic cancer. Our preliminary data yielded a high local control rate. Because of its ability to irradiate multiple targets simultaneously, helical tomotherapy could be an ideal palliative option for challenging cases of pancreatic cancer with metastases. Further large scale clinical trials are needed to verify the efficacy and safety of helical tomotherapy with concurrent capecitabine for treating advanced pancreatic cancer. Also, careful selection of those patients that stand to benefit from this regimen is needed.

## Competing interests

The authors declare that they have no competing interests.

## Authors' contributions

JJ participated in data collection, performed the statistical analysis and drafted the manuscript. CH conceived of the study, and participated in its design and coordination. JJ participated in data collection and helped to draft the manuscript. JK helped in data collection and analysis. YK helped in data collection and drafted the manuscript. BL, BK, HC, CK and IC helped to data analysis and drafted the manuscript. All authors read and approved the final manuscript.
